# Evolution of viral variants in remdesivir‐treated and untreated SARS‐CoV‐2‐infected pediatrics patients

**DOI:** 10.1002/jmv.27285

**Published:** 2021-09-04

**Authors:** Florencia A. T. Boshier, Juanita Pang, Justin Penner, Matthew Parker, Nele Alders, Alasdair Bamford, Louis Grandjean, Stephanie Grunewald, James Hatcher, Timothy Best, Caroline Dalton, Patricia Dyal Bynoe, Claire Frauenfelder, Jutta Köeglmeier, Phoebe Myerson, Sunando Roy, Rachel Williams, Thushan I. de Silva, Richard A. Goldstein, Judith Breuer, The COVID‐19 Genomics UK (COG‐UK) consortium

**Affiliations:** ^1^ Department of Infection, Immunity and Inflammation, UCL Great Ormond Street Institute of Child Health University College London London UK; ^2^ Division of Infection and Immunity University College London London UK; ^3^ Department of Infectious Disease Great Ormond Street Hospital for Children NHS Foundation Trust London UK; ^4^ Department of Infection, Immunity and Cardiovascular Diseases, The Florey Institute University of Sheffield Sheffield UK; ^5^ Department of Metabolic Medicine UCL Great Ormond Street Institute of Child Health London UK; ^6^ Department of Microbiology Great Ormond Street Hospital for Children NHS Foundation Trust London UK; ^7^ Department of Pharmacy Great Ormond Street Hospital for Children NHS Trust London UK; ^8^ Department of Ears Nose and Throat, Great Ormond Street Hospital for Children NHS Foundation Trust London UK; ^9^ Division of Surgery University of Adelaide Adelaide South Australia Australia; ^10^ Department of Gastroenterology Great Ormond Street Hospital for Children NHS Foundation Trust London UK

**Keywords:** intrahost, remdesivir, SARS‐CoV‐2, viral‐variants

## Abstract

Detailed information on intrahost viral evolution in SARS‐CoV‐2 with and without treatment is limited. Sequential viral loads and deep sequencing of SARS‐CoV‐2 from the upper respiratory tract of nine hospitalized children, three of whom were treated with remdesivir, revealed that remdesivir treatment suppressed viral load in one patient but not in a second infected with an identical strain without any evidence of drug resistance found. Reduced levels of subgenomic RNA during treatment of the second patient, suggest an additional effect of remdesivir on viral replication. Haplotype reconstruction uncovered persistent SARS‐CoV‐2 variant genotypes in four patients. These likely arose from within‐host evolution, although superinfection cannot be excluded in one case. Although our dataset is small, observed sample‐to‐sample heterogeneity in variant frequencies across four of nine patients suggests the presence of discrete viral populations in the lung with incomplete population sampling in diagnostic swabs. Such compartmentalization could compromise the penetration of remdesivir into the lung, limiting the drugs *in vivo* efficacy, as has been observed in other lung infections.

## 1 BACKGROUND

Severe acute respiratory syndrome coronavirus 2 (SARS‐CoV‐2), which causes coronavirus disease 2019 (COVID‐19), was first identified in Wuhan, China in December 2019. On March11, 2020, the WHO declared COVID‐19 a global pandemic. Since then, an estimated 50 million people have been infected, of whom up to 2.5% have died.^1^ A number of studies have assessed nasal and oropharyngeal viral load data from longitudinally sampled SARS‐CoV‐2‐infected patients. Their findings reveal wide variations in viral load at presentation.^2^, ^3^ However, milder disease and clinical recovery are associated with lower and declining viral load respectively, pointing to its potential use as a biomarker for antiviral drug response. Remdesivir, an RNA‐dependent‐RNA polymerase (RdRp) inhibitor, has been shown in one large randomized clinical trial (RCT) to be effective against SARS‐CoV‐2, although another large study, showed no clinical benefit and smaller studies have shown limited or no impact on clinical recovery.^2^, ^4^, ^5^, ^6^, ^7^, ^8^ Where clinical trial data are lacking or contradictory we have previously used deep pathogen sequencing, mutational analysis, and evolutionary modeling to gain insight into the impact of repurposed drugs, including RdRp inhibitors similar to remdesivir, on serious respiratory RNA viral infections in hospitalized patients.^9^, ^10^ These studies have revealed drug‐related mutational signatures, evidence of viral compartmentalization in the lung and previously unrecognized synergy between combination therapies associated with changes in viral loads and improved clinical outcomes. Here we report the application of similar methods in a personalized medicine approach to investigating the impact of remdesivir on SARS‐CoV‐2 within an individual and to evaluate potential biomarkers that can be used to monitor clinical efficacy. The data provide further insights into fundamental questions of SARS‐CoV‐2 evolution and coinfection.

## 2 METHODS

### 2.1 Sample collection and viral sequencing

Nasopharyngeal swab samples were collected and tested for SARS‐CoV‐2. Full‐length SARS‐CoV‐2 genome sequences were obtained from all positive samples using SureSelect^XT^ target enrichment and Illumina sequencing. For each patient, a unique patient reference was generated by mapping the remaining reads of the first sample to the SARS‐CoV‐2 reference genome (NC_045512) from GenBank using bwa‐mem.^11^ Reads from the subsequent samples of the same patient were mapped to this patient reference. Consensus sequences were aligned using MAFFT.^12^ Only genomes with more than 80% genome coverage and a mean read depth of 100 or above were included in downstream analysis.

### 2.2 Phylogenetic analysis

The maximum likelihood tree of the alignment was constructed using RAxML,^13^ with the GTR model and 1000 bootstrap replicates. All trees were rooted on the SARS‐CoV‐2 reference genome NC_045512.

### 2.3 Analysis and figure generation

Analysis was completed in R 3.6.1 using Rstudio 1.2. In general, data were processed using the tidyverse family of packages (v1.2.1). We employed the fisher.test in R to compare the count‐data of mutations for treated and untreated samples across all individuals. The Mann–Whitney–Wilcoxon test was implemented using the wilcox.test in R. We used Pearson's and Spearman's rank correlation for correlation analysis. This was done using the lm.test and cor.test function in the stats package in R.

### 2.4 Haplotype reconstruction

Haplotypes were reconstructed using HAplotype Reconstruction Of Longitudinal Deep Sequences (HaROLD) with default settings.^14^ HaROLD does not statistically support haplotypes from a single minority variant alleles (MVAs), we therefore constructed by hand the haplotypes for Patient B. In this case, the haplotype frequency was taken to be that of the single MVA.

### 2.5 Quantification of subgenomic RNA

We employed Periscope to detect subgenomic RNA (sgRNA).^15^ sgRNA is identified based on the detection of the leader sequence at the 5ʹend (5ʹ‐AACCAACTTTCGATCTCTTGTAGATCTGTTCT‐3ʹ) of the sequence. To optimize recovery, we excluded genomes with less than 90% coverage and less than 100 mean read depth (MRD).

### 2.6 Minority variant calling

Minority allele variants had to have a frequency of above 2% and with a minimum of four supporting reads identified at sites with a read depth of ≥5 using VarScan.^16^ Transient MVAs, which occurred at one time point in an individual, were discarded from the analysis.

### 2.7 Structural biology

The structure of the spike protein PDB 6XR8 was visualized using VMD. Mutations were modeled using the Swiss model.

### 2.8 Study approval

We sequenced SARS‐CoV‐2 samples routinely collected for clinical monitoring from children hospitalized for COVID‐19 in London between early March and mid‐May 2020. This study was approved by Great Ormond Street Hospital (Clinical Audit Number #2857) and PHE Research Ethics and Governance Group (REGG) (R&D NR0195).

## 3 RESULTS

### 3.1 Overview of patients

We analyzed nine hospitalized SARS‐CoV‐2 positive pediatric cases with a mean age at the time of infection of 4.7 years old (range 0–14 years old), who were repeatedly sampled during the course of their SARS‐CoV‐2 infection. A summary of their clinical features is shown in Table 1. Five patients had pre‐existing comorbidities associated with primary or secondary immunodeficiency (Table 1). Two patients (B and C) were admitted from the community, three patients (E, F, and I) were long‐term inpatients who had healthcare‐acquired SARS‐CoV‐2, and four (A, D, G, and H) were transferred within days of diagnosis of testing positive for SARS‐CoV‐2 from other hospitals for tertiary‐care treatment. Of the nine patients, four (A, D, G, and H), of whom only H was known to be immunocompromised, were admitted to the Pediatric Intensive Care Unit (PICU) (Figure S1). The remaining patients were cared for in the appropriate source isolation and were not colocated during their SARS‐CoV‐2 infection (Figure S1). Patients, A, D, and G received 8–10 days of remdesivir through a compassionate pediatric access program. Patients A and G received 200 mg loading dose followed by 100 mg daily and Patient D received 5 mg/kg (10 mg) loading dose and 1.25 mg/kg (2.5 mg) once daily (Table 1).

**Table 1:** Overview of patient clinical, treatment, sampling, and viral lineage data for all nine patients

Patient	Gender	Age, years	Ethnicity	Weight, kg	Diagnosis	Immune status	Admitted from (MM/YY)	Healthcare associated	Days from first recorded symptom to first positive	Remdesivir (Y/N) [days]	Days in ICU	No. of Sequenced Samples	Lineage	Previous studies
A	Female	9	Other	39	bronchiectasis with prior lobectomy	Immunocompetent	Other hospital (04/20)		3	Y [8]	12	9	B.1.1	^47^, ^48^, ^49^
B	Female	6	Asian	25.7	severe chronic illness including kidney disease	Immunocompromised	Other hospital (03/20)		Unknown	N	0	3	B.1.1	^47^, ^48^, ^49^
C	Male	9	Black African	52.2	lymphoblastic leukemia, on chemotherapy	Immunocompromised	Community (03/20)		0	N	0	5	B.2.1	^47^, ^48^, ^49^
D	Male	0	White	1.98	ex 32‐week prematurity, required inotropes and ventilation	Immunocompetent	Other hospital (03/20)		1	Y [10]	22	7	B.1.1	^47^, ^48^, ^49^, ^50^
E	Male	2	Other	12.4	Neuroblastoma on chemotherapy	Immunocompromised	Long‐term inpatient	Suspected	1	N	0	5	B.1.p16	^47^, ^48^, ^49^
F	Male	0	Unspecified	7.4	extreme prematurity, short gut, chronic lung disease	Likely immunocompromised	Long‐term inpatient	Suspected	0	N	0	2	B.2.1	^47^, ^48^, ^49^
G	Male	14	Unspecified	120	obese	Immunocompetent	Other hospital (04/20)		10	Y [10]	10	10	B.1.1.7	^47^, ^48^, ^49^
H	Male	1	Black African	10.3	Hepatoblastoma	Immunocompromised	Other hospital (04/20)		Unknown	N	34	13	B.1.p11	^47^, ^48^, ^49^
I	Female	1	Unspecified	9.85	PNET, on chemotherapy	Immunocompromised	Long‐term inpatient	Suspected	Asymptomatic	N	0	2	B.1.p16	^47^, ^48^, ^49^

### 3.2 Viral load trajectories by cycle threshold and clinical markers of infection

**Figure 1:**
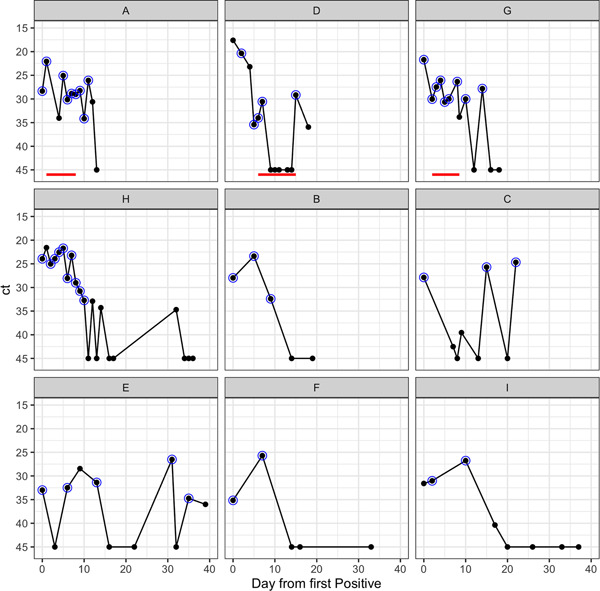
Ct trajectories of nine patients from 1st day up to 40 days post first positive. One panel per patient, red line indicates remdesivir received, black dot is sample taken, blue circle indicates sample successfully sequenced. Orange dot indicates bronchoalveolar lavage sample

Figure 1 shows the viral polymerase chain reaction (PCR) cycle threshold (ct) values for all nine patients for 40 days following their first positive sample available to us. Viral RNA was measured in nasopharyngeal aspirate for all patients and/or upper airway secretions for those who were intubated (Patients A, D, G, and H). Patient A also had a bronchoalveolar lavage. In agreement with earlier studies,^17^ the ct values showed a considerable day‐to‐day variation of between 0.16 and 14.4 cycle numbers (median 5.5 cycle numbers). Viral RNA continued to be detectable for 7 to over 50 days (median 16 days) following the first positive sample (Figure S2). Of the three patients who received remdesivir, only Patient D had total suppression of viral RNA during treatment followed by a rebound of the virus after treatment cessation (Figure 1). The four ICU patients were clinically most unwell, requiring assisted ventilation. All three remdesivir‐treated patients showed clinical improvement after starting the drug, associated with falls in temperature (all) and inflammatory markers (A and G) (Figure S3). All three were weaned from conventional ventilation before the treatment course was completed with decreases in oxygen requirements. In Patient D, a significant reversal of respiratory deterioration was noted once remdesivir was started; inhaled nitric oxide was stopped within 96 h coincident with weaning from high frequency oscillatory to conventional ventilation. Patient D, who alone required inotropic support, achieved hemodynamic stability off inotropes within 5 days of starting remdesivir.

### 3.3 Inter‐ and intrahost phylodynamics of SARS‐CoV‐2

**Figure 2:**
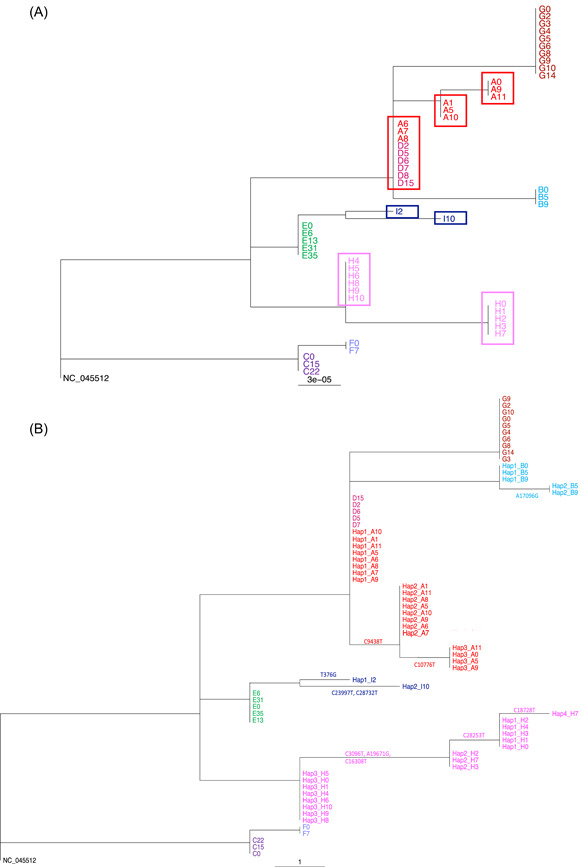
RAxML phylogenetic trees rooted at NC_045512. (A) Tree using consensus level sequences for Patients A–I. Boxes highlight distinct, identical sequences excluding gaps, found in Patients A, H, and I over time. Samples are labeled as [Patient][Time]. (B) Tree using haplotype sequences for Patients A, B, H, and I and consensus levels sequences for Patients D–G for which no haplotypes are identified. Haplotypes defining mutations are shown along the corresponding branches. Samples are labeled as Hap [number]_[Patient][Time]

To investigate the possibility of remdesivir resistance in Patients A, D, and G, we deep sequenced all samples as well as those from untreated patients for comparison (Figure 1). The sequencing metrics for the samples are summarized in Table S1. Relative to their first available sample, there were nine polymorphisms identified in viruses from Patients A, H, and I, of which five were nonsynonymous with four in the ORF1ab (nsps 1, 3, 4, 5) and one in the Spike protein, S2 subdomain (Table S2). None of the identified SNPs were sites identified as common homoplasies or those known to be susceptible to Illumina sequencing error, none have been associated with remdesivir resistance and none, other than spike mutation P812L, which is located within a predicted CD4 T cell epitope, were in known or predicted immune epitopes.^18^, ^19^, ^20^, ^21^, ^22^, ^23^, ^24^, ^25^ P812L was not predicted to alter spike protein structure, although it is not known whether it would abrogate T cell binding (Figure S4).^18^ Patient E was negative for SARS‐CoV‐2 in consecutive samples obtained on Days 16 and 22. The virus detected again at Days 31 and 35 was identical to all other sequences from this patient. We observed changes in the consensus sequences between sequential samples from Patients A, H, and I. Sequences A at time Points 6, 7, and 8 were identical to those of samples sequenced from patient D (Figure 2A). No evidence of laboratory contamination to explain the identity between A and D sequences was found.

### 3.4 No mutagenic signature identified for remdesivir

In vitro studies have not shown lethal mutagenesis to be a feature of remdesivir.^26^, ^27^ To exclude nonlethal mutagenesis as a possible explanation for continuing high viral RNAs despite remdesivir treatment, we compared the mutational burden and patterns of transitions and transversions in treated and untreated patients. In accordance with current understanding of its mode of action, we found neither an increased mutational burden in remdesivir‐treated patients nor any evidence of associated mutational signature (Figures S5 and S6). We found no evidence that the proportion of transitions and transversions distribution of mutation was dependent on the treatment (Fisher *t* test, *p* = 0.13).

### 3.5 Measurement of subgenomic RNA

**Figure 3:**
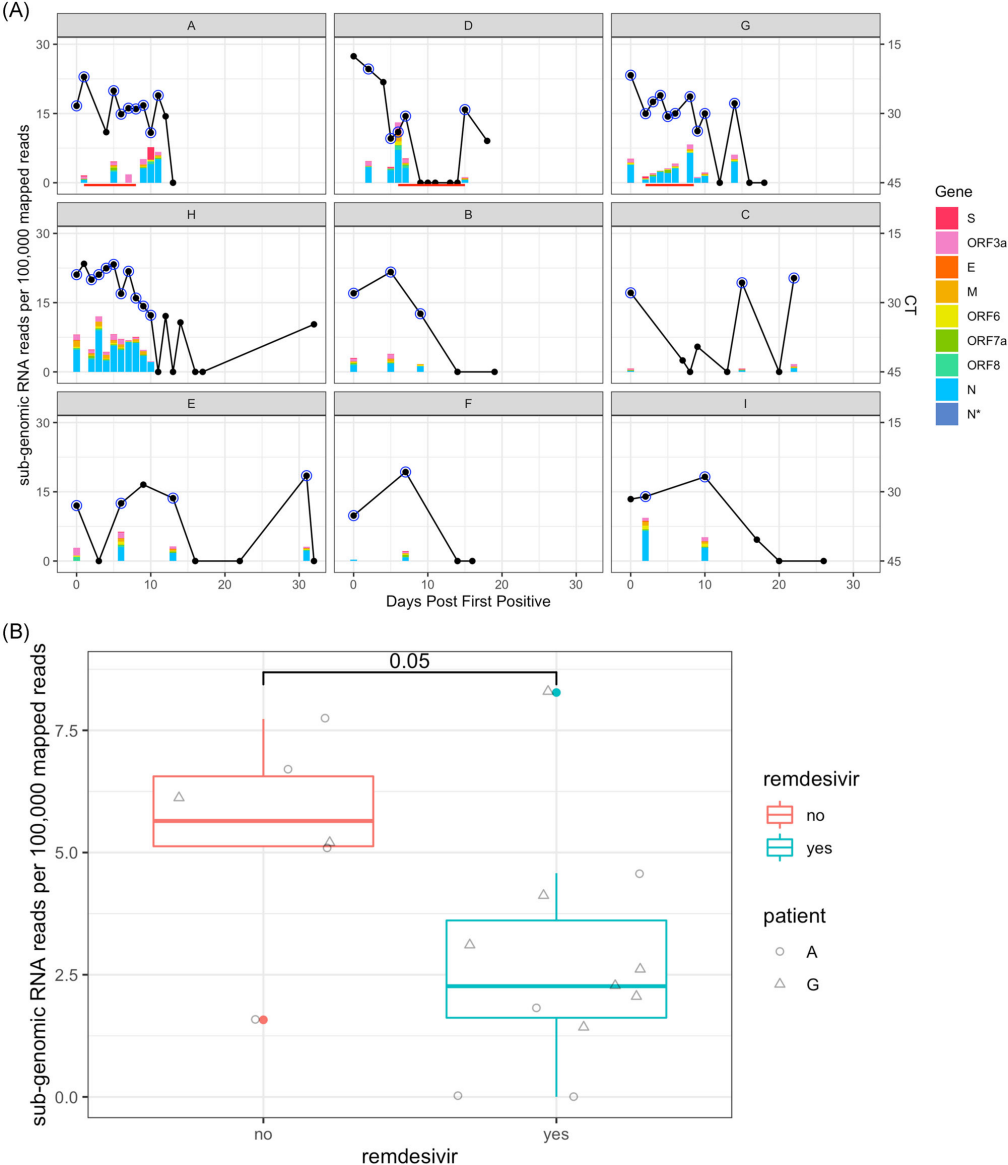
Evaluation of effect of remdesivir on levels of sgRPHT. (A) Comparison of ct values and sgRPHT over time by Patient. Stacked bars represent sgRPHT values colored by gene. Black line represent ct values, with blue circles annotating successfully sequenced samples. *Y*‐axis is days post first positive. (B) Box‐plot of sgRPHT on and off remdesivir for Patients A and G. Samples from each individual are identified by their shape. Treated samples have low sgRPHT than untreated samples taken in the same time‐window post first positive (Mann–Whitney–Wilcoxon test, *p* = 0.05). Patient D was excluded from this comparison as no sequences were available during remdesivir treatment as viral load was below the limit of detection

To determine whether, despite stable viral ct values, remdesivir treatment may have inhibited viral replication we analyzed subgenomic RNA (sgRNA) detected by the genome sequencing using Periscope.^15^ Stacked bar‐plots of the frequency of sgRNA reads per 100 000 mapped reads (sgRPHT) for each gene and corresponding ct values for each patient are compared in Figure 3A. Unlike previous reports, no correlation was found between sgRPHT and ct‐values (Figure S7A).^28^ We have excluded Patient D from the comparative analysis below as their virus was suppressed below the limit of detection during remdesivir treatment. sgRNA levels in samples taken during remdesivir treatment for Patients A and G were lower than in samples off treatment (Mann–Whitney–Wilcoxon test *p* = 0.05) (Figure 3B). Samples 6, 7, and 8 in Patient A had mean viral ct values of 28.7 (range 28.24–29.02) with barely detectable sgRNA despite high MRDs (3402‐6297). sgRNA was detected in all other samples with ct values <35 bar two. A similar tendency towards significance of remdesivir treatment on levels of sgRPHT was shown across all patients over all time points (Mann–Whitney–Wilcoxon test *p* = 0.059) (Figure S7B).

### 3.6 Evidence of mixed infection

**Figure 4:**
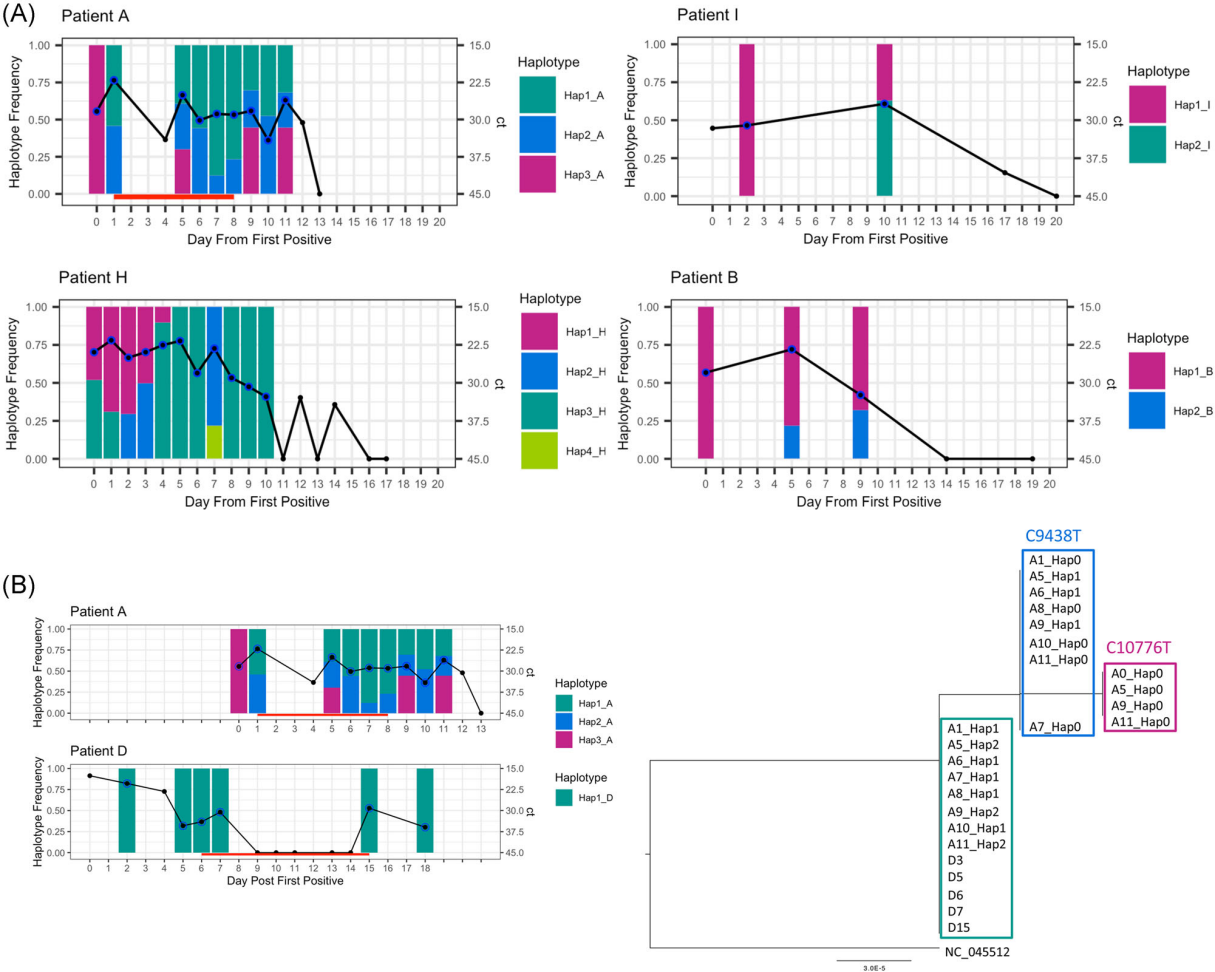
Frequency of identified haplotype over time for individual patients. (A) Haplotype frequency over time for Patients A, B, H, and I. (B) Right: phylogenetic tree of haplotypes from Patients A and D, nucleotide mutation shown above each cluster; left: haplotype frequency over time for Patients A and D. Black line is Ct value, red line indicates remdesivir received, black dot is sample taken, blue circle indicates sample successfully sequenced. Bars indicate frequency of identified haplotypes

We observed changes in the consensus sequences at different timepoints in Patients A, H and I (Figure 2A). As depicted on the tree, no SNPS relative to reference sequence NC_045512 are shared across all patients and neither are there any such SNPs that are unique to patients admitted to ITU and/or on treatment (Table S3). To further examine this and the identical sequences from Patients D and A, we analyzed MVAs as outlined in the methods. A complete list of identified polymorphisms for each sample, and corresponding frequency and read support, can be found in Table S4. Patients A, B, H, and I had well‐supported MVAs which varied in frequency over time (Figure S8). MVAs in other patients occurred for the most part on a single occasion or at levels less than 20% with poor read support. To resolve possible mixed infections within each sample, we used the haplotype reconstruction method HaROLD.^14^ HaROLD identified three haplotypes for Patient A, four for Patient H, two for Patient I, two in Patient B with one in all other patients. All identified haplotypes are labeled as Hap_[number]_[Patient][Time]. Haplotypes clustered phylogenetically by patient other than Hap1_A, found at the root of the clade from Patient A, which was identical to viral sequences from Patient D (Figure 2B). We observed no obvious pattern of haplotype change in any of the patients, with sample‐to‐sample variation in viral loads and haplotype abundance occurring particularly in Patients A and H (Figure 4A).

We next investigated whether the haplotypes within Patients A, H, I, and B were likely to represent coinfections with different viruses.^29^ Using Local Lineage and Monophyly Assessment (LLAMA),^30^ we identified the nearest samples to each haplotype in the global alignment on COVID‐19 Genomics UK (COG‐UK) consortia.^31^ The local trees identified by LLAMA are shown in Figure S9. Comparison of haplotypes in Patients A, H, and I with global sequences confirmed that haplotypes Hap1_A, Hap2_A, Hap3_H, and Hap1_I were circulating independently globally as were the single genotypes from Patients C, E, and F (Figure S9). However, haplotypes Hap3_A, Hap1/2_B, Hap1/2_H, and Hap2_I as well as the virus from Patient G were not represented among 61740 global sequences available on GISAID.^32^ This may reflect incomplete population sampling or the recent emergence of new lineages. Alternatively, it is possible that some haplotypes are not currently freely circulating in the population and possibly that the mutations they carry are deleterious as has been postulated for influenza.^33^ The close clustering between each of the noncirculating viruses with other haplotypes in their cognate hosts supports within‐host evolution (Figure S9), as opposed to coinfection with multiple viruses as has previously been suggested.^34^

A possible exception to this conclusion was Patient A. It is conceivable that haplotype Hap1_A, which is identical to the viral sequence from patient D, could have resulted from superinfection. Patients A and D were colocated in the Intensive Care Unit (ICU) (Figure S1). Haplotype Hap1_A was not identified in the sample taken from Patient A before their transfer to the ICU but was detected approximately 28 h later in a sample taken 2 h before starting remdesivir (Figure 4B). However, no other healthcare‐associated transmissions were reported in the ICU and local epidemiological investigation suggests it to have been unlikely. Moreover, in view of the rapid appearance of Hap1_A following transfer to ICU and the variation in haplotype frequencies over time, it is more probable that Hap1_A and 2_A were present in the patient's first sample but not detected. This would support the within‐host evolution of these three closely related strains, for one of which, Hap3_A, there is no evidence of circulation in the population.

## 4 DISCUSSION

Our comparative analysis of longitudinal samples from remdesivir‐treated and untreated patients infected with SARs‐CoV‐2 identifies evidence of remdesivir‐associated suppression of viral RNA and sgRNA *in vivo* and uncovers the presence of mixed viral haplotypes likely to have evolved within each host early in infection, persisting thereafter, in some cases possibly within discrete tissue compartments in the lung.

Although remdesivir may have resulted in clinical improvements for the patients described here, only in Patient D was this accompanied by a fall in viral RNA levels. Viral RNA remained undetectable until treatment was stopped, when it again rose. In Patient A, no change in viral RNA levels occurred during remdesivir treatment; however, SARS‐CoV‐2 sgRNA levels appear to have reduced, increasing again following cessation of treatment (Figure 4). Although sgRNA levels have been shown to have a weak association with viral replication in in vitro culture,^35^ the extent to which sgRNA levels reflect viable virus better than viral RNA remains controversial.^28^ Our data samples obtained during remdesivir therapy had lower sgRNA levels even when high viral RNA persisted (Figure 4). The samples have good coverage and MRDs (Table S1), thus excluding RNA degradation as the cause of low sgRNA. Our results mirror findings from remdesivir study in the macaques model which showed viability in in vitro culture of virus from upper respiratory samples is decreased and clinical scores are improved, despite no change in viral RNA levels.^36^ The possibility that sgRNA, may, together with viral load, provide a biomarker of response to remdesivir should now be explored.

We observe considerable variation in the consensus SARS‐CoV‐2 sequences obtained from four of the nine patients, which appears unrelated to remdesivir treatment. The heterogeneity is explained by the presence of between 2 and 4 stable viral haplotypes that vary in abundance (Figure 4A). From this, we see that, while remdesivir suppressed viral replication in Patient D, the same dose had little impact on the viral RNA from the identical strain, Hap1_A, in Patient A (Figure 4B). Remdesivir resistance was not found to account for the difference in response. Instead, the variable abundance of multiple distinct haplotypes present in different samples from Patients A, B, H, and I, including in samples obtained from deep within the lung, is consistent with the presence of tissue compartmentalization, wherein a pathogen replicates in physically separated niches within the lung potentially accumulating mutations that allow different populations to be distinguished. Diagnostic specimens that incompletely sample these poorly mixed virus populations, result in mixed haplotypes that vary in frequency from sample‐to‐sample, as demonstrated in our patient data. The findings are supported by the independent reports that SARS‐CoV‐2 viral loads vary in different areas of lung sampled postmortem.^37^

Tissue compartmentalization is well described for other inflammatory lung infections including *Mycobacterium tuberculosis* and influenza and has been associated with uneven drug penetration, leading to poor resolution of infection and predisposing to drug resistance.^10^, ^38^, ^39^ Modeling of drug levels within lung tissue from SARS‐CoV‐2‐infected patients also suggests that remdesivir penetration into lung tissue is poor.^40^ In Patient A, the persistence of different viral populations at variable frequencies, including in bronchoalveolar lavage, supports the presence of viral compartmentalization as potentially contributory to reduced remdesivir effect. The rebound of viral sgRNA and RNA at the end of remdesivir in Patients A and D, respectively also suggests a suboptimal duration of treatment (Figures 1 and 3A). At the same time suppression of viral RNA in Patient D may reflect higher remdesivir tissue levels seen in neonates in which drug clearance is reduced.^41^ Taken together with pharmacokinetic‐pharmacodynamic (PKPD) models of SARS‐CoV‐2 viral dynamics, which predict that greater than 90% inhibition of replication is required to interrupt viral replication, the results presented here corroborate predictions that remdesivir (87% inhibition) monotherapy is unlikely to succeed.^42^ Based on previous experience of treating serious RNA infections in the lung, we speculate that early treatment with combination therapy may be required for antiviral effect.

The haplotypes we constructed for Patients A, B, H, and I were phylogenetically sister taxa, with generally only one haplotype per patient identical to freely circulating lineages (Figure S9). The only outlier in this respect is Patient A, which shared a haplotype with Patient D. It is possible that Patient A could have acquired their haplotype through cotransmission or superinfection.^34^ However, on balance, the data do not support this. Patients B, H and I were known to be immunocompromised, while Patient A remains under investigation for immune dysfunction. Within‐host evolution of stable viral haplotypes (clones) is well described for norovirus and influenza in immunocompromised patients.^43^, ^44^ Moreover, evolution of variants in SARS‐CoV‐2 has also been described in several immunocompromised patients, although in the absence of haplotype reconstruction, whether these represent multiple co‐existing genotypes is unclear.^45^ We, therefore, conclude that early within‐host evolution most logically explains the diversity seen in Patients A, B, H, and I.

A major caveat to our findings is that of our cohort pediatric, for which both the clinical picture and outcome of SARS‐CoV‐2 infections are known to differ from adults. Notwithstanding, similar patterns of clinical and virological response to remdesivir have been described in adults.^2^, ^7^, ^46^ Moreover, both clinical and viral sequence data from the use of repurposed drugs to treat other severe RNA viral infections have shown similarities in adults and children.^10^, ^38^ Additionally, although the largest of its kind, our cohort is small, with interpatient clinical and genomic heterogeneity patterns restricting some of the conclusions that can be drawn. Nevertheless, some observations, such as the observation of persistent viral subpopulations, span several patients with different clinical prognoses. Larger studies using deep clinical and viral profiling of multiple samples from adult patients treated with remdesivir alone and in combination would provide better insight.

In summary, we show that treatment with remdesivir is capable of suppressing SARS‐CoV‐2 viral and subgenomic RNA *in vivo* and demonstrate that the latter, in particular, needs further investigation as a potential biomarker for monitoring antiviral therapy. Our data suggest that heterogeneous response to remdesivir is not due to resistance but rather is likely to be caused by suboptimal tissue levels. The patterns of SARS‐CoV‐2 within‐host genetic heterogeneity uncovered by deep sequencing may be most parsimoniously explained by viral compartmentalization within lung‐tissue, a factor that is already known to impede drug penetration in patients with other lung infections. This may compound inherently poor remdesivir tissue penetration and rapid clearance of active metabolites in those with normal renal function. We and others have shown that where compartmentalization occurs in influenza and *M. tuberculosis*, combination therapies improve outcomes.^10^, ^38^ Based on our experience of using similar drugs for the treatment of serious RNA viral infections, we propose that a more personalized medicine approach combining *in vitro* and *in vivo* pharmacokinetic measurements, viral RNA, and sgRNA profiling together with viral evolutionary modeling could help to optimize the use of remdesivir both alone and in combination for treatment of SARS‐CoV‐2.

## The COVID‐19 Genomics UK (COG‐UK) Consortium

A full list of consortium names and affiliations can be found at https://www.cogconsortium.uk.

## CONFLICT OF INTERESTS

The authors declare that there are no conflict of interests.

## AUTHOR CONTRIBUTIONS

Florencia A. T. Boshier and Juanita Pang wrote the manuscript, generated all figures, selected and incorporated all analysis, merged all datasets, and processed all the genomic data. Order was assigned according to seniority. Justin Penner collected clinical metadata across the course of individuals infections and interpretation of clinical significance of study. James Hatcher provided access to complementary metadata for subset UK sequences and supported phylogenetic analysis. Matthew Parker instructed on implementation and interpretation of PERISCOPE. Nele Alders, Alasdair Bamford, Louis Grandjean, Stephanie Grunewald, James Hatcher, Timothy Best, and Caroline Dalton provided treatment information for individuals as well as interpretation of clinical significance of study. Patricia Dyal Bynoe extracted all clinical samples. Claire Frauenfelder and Jutta Köeglmeier provided additional treatment information for individuals. Phoebe Myerson carried out structural biology analysis. Sunando Roy sequenced all samples. Rachel Williams managed the extraction and sequencing of samples. The COVID‐19 Genomics UK (COG‐UK) consortium funded and managed the extraction and sequencing of all UK sequences. Thushan I. de Silva instructed on implementation and interpretation of PERISCOPE. Richard A. Goldstein designed and oversaw phylogenetic analysis. Judith Breuer conceived the study and oversaw all analysis undertaken. All authors contributed to the revisions of the manuscript.

## Supporting information

Supporting information.

Supporting information.

Supporting information.

Supporting information.
